# The *Vibrio cholerae* diguanylate cyclase VCA0965 has an AGDEF active site and synthesizes cyclic di-GMP

**DOI:** 10.1186/1471-2180-14-22

**Published:** 2014-02-04

**Authors:** Jessica L Hunter, Geoffrey B Severin, Benjamin J Koestler, Christopher M Waters

**Affiliations:** 1 Department of Microbiology and Molecular Genetics, Michigan State University, East Lansing, MI 48824, USA; 2 BEACON Center for the Study of Evolution in Action, Michigan State University, 5180 BPS 567 Wilson Road, East Lansing, MI 48824, USA

**Keywords:** Diguanylate cyclase, Phosphodiesterase, Cyclic di-GMP, Biofilm, GGDEF

## Abstract

**Background:**

Diguanylate cyclases (DGCs) regulate biofilm formation and motility in bacteria by synthesizing the second messenger cyclic di-GMP (c-di-GMP) in response to environmental stimuli. DGC enzymatic activity is believed to be dependent on the presence of a GG(D/E)EF active site motif, however approximately 25% of known DGCs contain a degenerate active site. The *Vibrio cholerae* protein VCA0965 contains an AGDEF active site and is presumed to be an inactive DGC.

**Results:**

Ectopic expression of VCA0965 in *V. cholerae* causes a 3-fold reduction in flagellar-based motility. Additionally, an RXXD allosteric inhibition mutant of VCA0965 strongly inhibited motility and stimulated biofilm formation. This activity was lost when the active site of VCA0965 was mutated to AGDAF, suggesting that VCA0965 synthesizes c-di-GMP. In support of this, ectopic expression of VCA0965 and VCA0965 containing a mutation in its RXXD motif significantly increased the intracellular c-di-GMP levels in *V. cholerae* and *Escherichia coli*. Furthermore, we found that purified VCA0965 was able to synthesize c-di-GMP *in vitro*. Systematic mutation of the first amino acid in the AGDEF motif of VCA0965 revealed that glycine, methionine, and histidine also produced an active DGC capable of inhibiting motility and increasing the intracellular concentration of c-di-GMP in *V. cholerae*.

**Conclusions:**

Based on these results, we conclude that VCA0965 is capable of c-di-GMP synthesis and that the first amino acid of the GG(D/E)EF motif is more tolerant of substitutions than currently appreciated.

## Background

Cyclic di-GMP (c-di-GMP) is a bacterial second messenger that is widely utilized by bacteria, with more than 80% of sequenced bacteria predicted to use this signal [1,2]. C-di-GMP controls a variety of phenotypes, including biofilm formation, motility, and virulence in multiple bacteria [3-8]. In *Vibrio cholerae*, high levels of c-di-GMP induce biofilm formation, reduce swimming motility, and inhibit colonization in a murine infection model [9,10]. These c-di-GMP associated behaviors are enacted through c-di-GMP binding to a variety of receptor proteins [5,11-13] and, potentially to one of two classes of riboswitches [14,15]. Intracellular levels of c-di-GMP are controlled through synthesis from two molecules of GTP by diguanylate cyclases (DGC) [16] and degradation to pGpG or GMP by phosphodiesterases (PDE) [6,17].

DGCs are characterized by the presence of a conserved GGDEF domain composed of approximately 200 amino acids [18]. These domains are believed to require the specific amino acid sequence GG(D/E)EF in their active site (referred to as A-site here) in order to retain their enzymatic activity. In addition to their active site motif, approximately 53% of GGDEFs contain an RXXD motif [1]. The RXXD motif is a feedback inhibition site (referred to as the I-site here) located near the active site, which specifically binds to dimeric c-di-GMP to non-competitively inhibit enzyme activity [19]. EAL domains are one of the two enzymatic domains that contain c-di-GMP specific phosphodiesterase (PDE) activity, the other being the HD-GYP domain [6,17]. Approximately 67% of DGCs are multi-domain proteins, containing at least one partner domain, with the most common partner domain being the EAL domain [1]. Interestingly, in more than 40% of these EAL-GGDEF proteins (and in 24% of DGCs in general) the GGDEF active site is degenerate at one or more amino acids, suggesting that many of these proteins are incapable of c-di-GMP synthesis [1].

One explanation for the high frequency of degenerate DGCs is that these proteins in some cases act as c-di-GMP receptors by binding to c-di-GMP at either their degenerate active site or their RXXD inhibition site. These degenerate DGCs respond to c-di-GMP in several ways. C-di-GMP may mediate participation of the DGC in a regulatory cascade, as does PopA from *C. crescentus*, PelD from *Pseudomonas aeruginosa*, and CdgA from *Bdellovibrio bacteriovorus* which bind c-di-GMP to regulate cell cycle progression, biofilm formation, and predation, respectively [20-22]. Binding of c-di-GMP may be required for proper localization of the protein akin to both FimX from *P. aeruginosa* and SgmT from *Myxococcus xanthus*[23,24]. Additionally, degenerate DGCs may also retain other roles independent of c-di-GMP binding. CdpA in *V. cholerae* requires its degenerate GGDEF domain, but not c-di-GMP, to retain its PDE activity, and the highly degenerate GGDEF domain of YybT from *Bacillus subtilis* exhibited ATPase activity [10,25]. In one case, a degenerate DGC has been shown to be active as the *Pectobacterium atrosepticum* DGC ECA3270 retains DGC activity despite having a degenerate SGDEF active site motif [26]. Interestingly, evidence is accumulating that DGCs and PDEs themselves form protein complexes, and it is intriguing to speculate degenerate DGCs impact these processes [27,28].

Here we investigate the degenerate *V. cholerae* DGC VCA0965. We examined the ability of all 40 *V. cholerae* DGCs to inhibit motility in semisolid agar, and we determined that VCA0965 was active in this assay. This result was surprising as VCA0965 is a DGC that encodes a degenerate AGDEF active site. Rather than functioning as a receptor for c-di-GMP, our results suggest that VCA0965, despite its degenerate active site motif, is capable of c-di-GMP synthesis.

## Methods

### Bacterial strains and culture conditions

Strains used in this study are listed in Table 1. Site directed mutants were generated using QuikChange Lightning Site-Directed Mutagenesis Kit (Agilent Technologies) according to the manufacturer’s protocols. *E. coli* and *V. cholerae* strain C6706str2 were grown in LB at 35°C with shaking at 220 rpm unless otherwise indicated. LB was supplemented with 100 μg mL^-1^ kanamycin as needed to maintain plasmids. Plasmids were induced with 0.1 mM isopropyl-β-D-thiogalactoside (IPTG) unless otherwise specified.

**Table 1:** Strain and plasmid list

**Strain or plasmid**	**Relevant features**	**Source or citation**
**Strain**		
*Vibrio cholerae*		
C6706str2	Wild type strain	[29]
CW2034	Δ*vpsL*	[32]
**Plasmid**		
pEVS141		[30]
pCMW75	pEVS143 backbone, overexpression of QrgB (*Vibrio harveyi* DGC) under Ptac promoter	[31]
pVCA0965	pEVS143 backbone, overexpression of VCA0965 under Ptac promoter	[30]
pVCA0965-A Site	pEVS143 backbone, overexpression of VCA0965 E287A under Ptac promoter	This study
pVCA0965-I Site	pEVS143 backbone, overexpression of VCA0965 R267A, D270A under Ptac promoter	This study
pVCA0965-AI Site	pEVS143 backbone, overexpression of VCA0965 R267A, D270A, E287A under Ptac promoter	This study
pVCA0965(180–396)	pET28b backbone, overexpression of VCA0965 (180–396) with a 6 histidine C terminal tag	This study

### Motility assay

**Figure 1:**
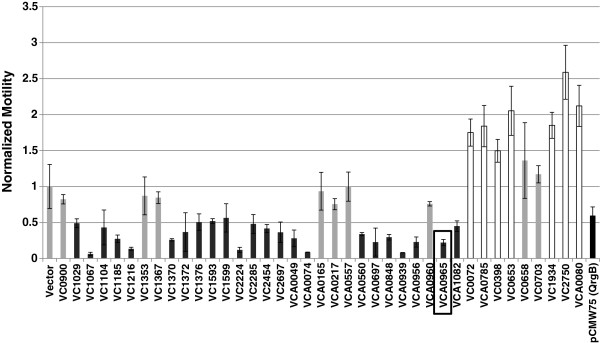
**Motility repression by *Vibrio cholerae *GGDEFs.** The swimming motility of *V. cholerae* ectopically expressing each of its GGDEF proteins was determined in 0.5% agar plates. Motility is normalized to the vector control, and the mean and standard deviation are shown. 23 GGDEF proteins significantly reduced motility (p < 0.05, black bars), while 7 others significantly increased motility (p < 0.05, white bars). Gray bars are not significantly different from the vector control. VCA0965 is highlighted in the black box, and QrgB is a DGC from *V. harveyi* known to be active in *V. cholerae*.

Motility plates (60 mL) were prepared immediately before each assay from LB supplemented with 0.35% agar. Plates, supplemented with 100 μg mL^-1^ kanamycin and 0.1 mM isopropyl-β-D-thiogalactoside (IPTG) as appropriate, were allowed to solidify for 1 to 4 hours prior to use. Plates were inoculated from overnight cultures by stabbing through the agar with a pipette tip, and left undisturbed at room temperature for 1 hour before inversion and incubation at 37°C for 20 hours. Motile bacteria were digitally imaged using an Alpha Innotech Red Gel Imager with white light and an 800 ms exposure time. Images were processed to calculate colony area by determining differential pixel density using ImageJ software [31]. A sticker of known area was included on each plate for normalization to obtain the colony area in cm^2^. The initial motility screen (Figure 1) was conducted as above using 0.5% agar.

### Minimum biofilm eradication concentration (MBEC) and flow cytometry biofilm assays

MBEC plates were prepared by inoculating 150 μL of LB/well with a 1 to 1000 dilution from overnight *V. cholerae* cultures. Wells were supplemented with 100 μg mL^-1^ kanamycin and 0.1 mM IPTG as needed. Plates were incubated at 37°C with gentle aeration for 7 hours before washing in PBS, fixing with 95% ethanol and staining with 0.41% crystal violet in 10% ethanol. Crystal violet was eluted in 160 μL of 95% ethanol. The optical density at 600 nm (OD_600_) was determined using a Spectra Max M5 spectrometer (Molecular Devices). To avoid saturation, the elutions were also diluted 1 to 10 and the resultant OD_600_ was measured. If the OD_600_ of the undiluted elution exceeded 1, then the OD_600_ was calculated from the 1 to 10 dilution. Flow cytometry cultures were prepared by diluting an overnight culture 1:100 in LB. Cultures were supplemented with kanamycin and IPTG as needed and grown at 35°C and 220 rpm for 4 hours. Flow cytometry analysis was performed as described [32].

### Determination of the intracellular concentration of c-di-GMP

Cultures were prepared by diluting an overnight culture of *V. cholerae* 1:1000 into LB and grown to an OD_600_ between 0.6 and 1.0. Cultures were supplemented with kanamycin and IPTG as needed. Samples were extracted and analyzed by liquid chromatography coupled with tandem mass spectromentry (LC-MS/MS) as previously described [32] using an Acquity Ultra Performance liquid chromatography system (Waters) coupled with a Quattro Premier XE mass spectrometer (Waters). The concentration of c-di-GMP was determined by quantifying an 8-point standard curve of chemically synthesized c-di-GMP (Biolog) ranging from 1.9 nM to 250 nM.

### Protein purification

An overnight culture of *E. coli* JM109(DE3) (Promega) containing the pVCA0965(180–396) vector was prepared and used to inoculate LB broth supplemented by 100 μg mL^-1^ kanamycin. The culture was grown at 37°C with shaking until an OD_600_ between 0.7-0.8 was reached. The cells were then induced by addition of IPTG to a concentration of 1 mM. The induced cells were incubated between 16 and 18 hours at 16°C with shaking. Following induction, cells were collected via centrifugation in a Sorvall RC-5B Superspeed Centrifuge for 10 minutes at 2,678 g and 4°C. The pellet was then resuspended in lysis buffer (25 mM Tris-Cl (pH 8.0), 500 mM NaCl, 5 mM 2-mercaptoethanol, 20 μg/mL DNase, one tablet Roche protease inhibitor kit) and lysed using a M-110P processor (Microfluidcs) 3 times at 20,000 psi. Novagen Ni-NTA His-bind resin was equilibrated in binding buffer (25 mM Tris-Cl (pH 8.2), 500 mM NaCl, 20 mM imidazole, 5 mM 2-mercaptoethanol) and added to the lysate. Resin and lysate were incubated for 60 minutes at 4°C with constant end over end rotation. After incubation, lysate and resin were applied to a column and allowed to flow to pack. The resin was washed three times with small amounts of binding buffer. The protein was then step eluted by the addition of imidazole elution buffer (25 mM Tris-Cl (pH 8.2), 500 mM NaCl, 20 mM imidazole, 5 mM 2-mercaptoethanol) with 50 mM, 100 mM, 200 mM, or 300 mM imidazole. VCA0965 was determined to elute in the 200 mM and 300 mM imidazole fractions. These fractions were concentrated on Amicon Ultra Centrifugal Units by centrifugation at 16,000 g for 20 minutes at 4°C. Concentrated fractions were washed once with dialysis buffer (30 mM Tris-Cl (pH 7.6), 100 mM NaCl) and then stored at −80°C in 20% glycerol.

### Determination of DGC activity

Purified proteins were diluted 1/25 and mixed with 0.015 mM GTP in reaction buffer (41 mM NaCl, 24 mM Tris-Cl, 1.25 mM MgCl_2_) for 24 hours at room temperature. Samples were then boiled for five minutes until precipitate formed and centrifuged at 15,871 g for 10 min. to remove precipitated proteins. The concentration of c-di-GMP was determined using LC-MS/MS as described above.

## Results

### Census of DGCs that regulate motility in *Vibrio cholerae*

*Vibrio cholerae* strain C6706str2 encodes 40 GGDEF domain containing proteins which potentially function as DGCs. As c-di-GMP represses motility in *V. cholerae*[33], we determined which of the 40 GGDEFs repress motility in laboratory conditions by individually overexpressing each from a Ptac promoter on a plasmid and measuring flagellar based swimming motility in 0.5% agar plates. We chose to survey the activity of all DGCs using a heterologous expression strategy, rather than mutagenesis analysis, because this approach ensures that each DGC is expressed in the conditions examined. Our results indicated that 23 of the GGDEF encoding proteins significantly repressed motility while 7 significantly increased motility (Figure 1). All 7 of the proteins that increase motility contain both a GGDEF domain and an EAL domain. Therefore, we hypothesize that these proteins likely possess net PDE activity resulting in decreased intracellular c-di-GMP. Interestingly, VCA0965 repressed motility 4.6-fold, yet it is predicted to be enzymatically inactive due to a non-canonical AGDEF active site. For this reason, we sought to further understand the role of VCA0965 in the regulation of c-di-GMP controlled behaviors in *V. cholerae.*

### The active site of VCA0965 is required for motility inhibition

**Figure 2:**
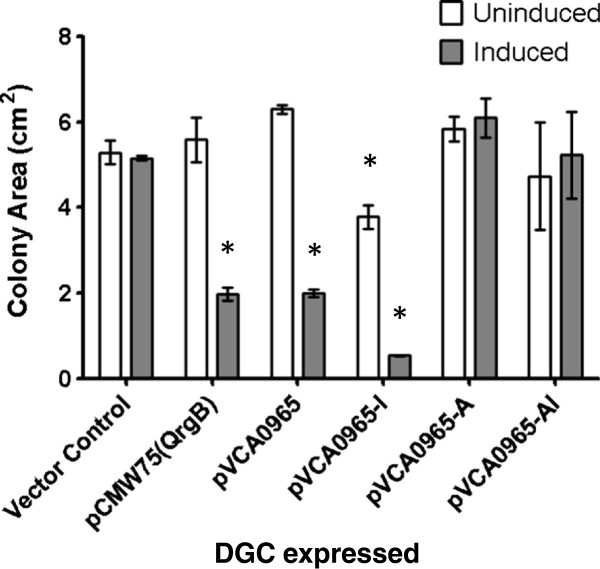
**VCA0965 requires an intact active site to repress motility.** Quantification of motility as determined by quantifying colony area (cm^2^) in *V. cholerae* strains expressing the indicated proteins from a Ptac promoter on a plasmid. Proteins were induced with 0.1 mM IPTG (gray bars). The mean and standard deviation are indicated (* = p < 0.05, n = 3). In this and all subsequent figures the strain designations are pCMW75 (QrgB) (an active DGC from *V. harveyi*), pVCA0965 (WT VCA0965 expression plasmid), pVCA0965-I (I-site mutant), pVCA0965-A (A-site mutant), and pVCA0965-AI (I-site/A-site double mutant).

We hypothesized that VCA0965 was incapable of synthesizing c-di-GMP and functioned as a c-di-GMP receptor protein to repress motility. Consistent with previously studied DGC effectors, c-di-GMP could bind VCA0965 at its degenerate active site (referred to as the A-site) and/or its RXXD allosteric inhibition site (referred to as the I-site) [10,20,21]. To determine if the A-site and/or I-site were necessary for motility repression, we mutated the VCA0965 I-site from RXXD to AXXA (VCA0965-I) and the VCA0965 A-site from AGDEF to AGDAF (VCA0965-A). We also constructed an allele of VCA0965 which contained both the A-site and the I-site mutations (VCA0965-AI). These mutant VCA0965 alleles were ectopically expressed from a plasmid in *V. cholerae* and then tested for the ability to repress swimming motility (Figure 2). Confirming our previous findings, ectopic expression of WT VCA0965 repressed motility 3-fold in a motility assay, as did the positive control QrgB, a DGC from the related species *Vibrio harveyi* that has been shown to be active in *V. cholerae*[34] (Figure 2). The I-site mutant more robustly repressed motility than WT VCA0965. This result demonstrated that the I-site is not required for motility repression. However, the A-site was necessary for this regulation as expression of both the VCA0965-A and VCA0965-AI proteins failed to inhibit motility (Figure 2).

### VCA0965-I induces biofilm formation

**Figure 3:**
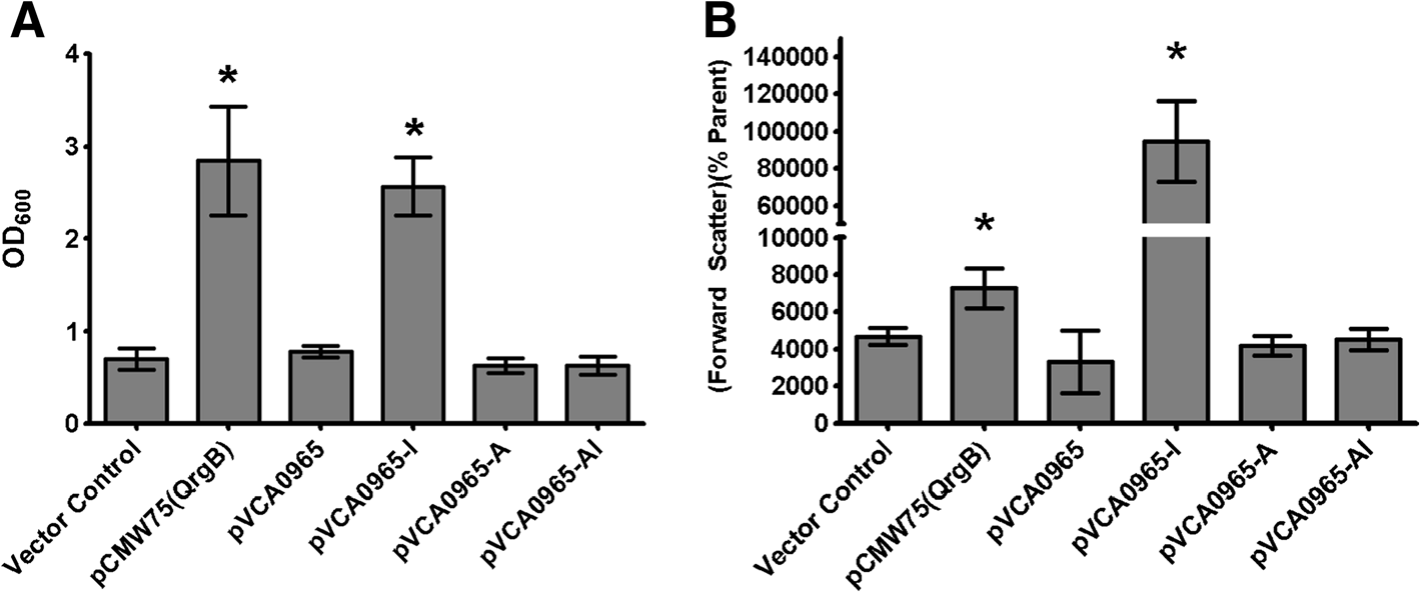
**Overexpression of the VCA0965-I mutant induces biofilm formation in an A-site dependent manner. (A)** Mean biofilm formation of *V. cholerae* overexpressing wild type VCA0965 and its mutant derivatives were measured using a MBEC assay. **(B)** Biofilm formation as measured by flow cytometry was determined by multiplying the mean forward scatter value of the aggregate population by the percentage of aggregates relative to the total events measured. The mean and standard deviation are indicated (* = p < 0.05, n = 3).

Like many bacterial species, biofilm formation in *V. cholerae* is induced by increased intracellular c-di-GMP [9]. As ectopic expression of VCA0965 was able to repress motility, we determined whether ectopic expression of VCA0965 would induce biofilm formation. Biofilm formation was quantified using two approaches. First we measured biofilms with the minimum biofilm eradication concentration (MBEC) biofilm assay. Expression of the positive control DGC, QrgB, induced biofilm formation four fold above the vector control (Figure 3A). Although overexpression of VCA0965 significantly inhibited motility, overexpression of VCA0965 did not affect biofilm formation. This result could be due to high-specificity signaling or differential activity of the DGC in these two experiments (see Discussion). In contrast, overexpression of VCA0965-I stimulated a 3-fold increase in biofilm formation (Figure 3A). Analogous to our results measuring motility, overexpression of VCA0965-A and VCA0965-AI did not induce biofilms. This result indicated that the ability of the I-site mutant to induce biofilm formation was dependent on the presence of an intact active site motif.

To confirm these results, we measured biofilm formation using flow cytometry as previously described [30]. This assay measures aggregate formation in liquid cultures, which is driven by extracellular polysaccharide production. Analysis of biofilms by flow cytometry was indeed similar to the results of the MBEC assay in that WT VCA0965 was unable to stimulate aggregate formation, but the VCA0965-I mutant strongly stimulated aggregate formation in an A-site dependent manner (Figure 3B).

### VCA0965 synthesizes c-di-GMP

**Figure 4:**
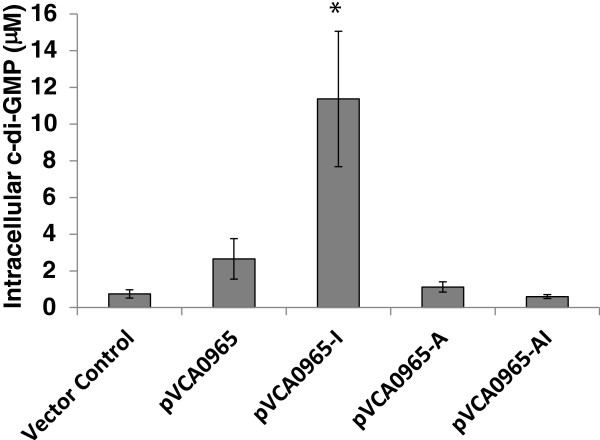
**VCA0965 increases the intracellular concentration of c-di-GMP.** Wild type VCA0965 and its mutant derivatives were overexpressed in *V. cholerae* grown to an OD_600_ of 0.6-1.0, and the resultant c-di-GMP levels were measured using LC-MS/MS. The mean and standard deviation are indicated (*= p < 0.05, n = 3).

The above findings that VCA0965 and/or its I-site mutant were able to repress motility and induce biofilm formation only with an intact AGDEF active site suggested that VCA0965 was capable of influencing intracellular c-di-GMP levels. To test this hypothesis, we measured the impact of VCA0965, VCA0965-I, VCA0965-A and VCA0965-AI overexpression on intracellular c-di-GMP levels in *V. cholerae* using liquid chromatography coupled with tandem mass spectrometry (LC-MS/MS). We observed that overexpression of VCA0965 and VCA0965-I caused a 4- and 15-fold increase, respectively, in the intracellular concentration of c-di-GMP compared to a vector control (Figure 4). Neither pVCA0965-A nor pVCA0965-AI caused a significant change in c-di-GMP levels when overexpressed, as would be expected if VCA0965 possessed DGC activity (Figure 4). We obtained similar results if these variants of VCA0965 were expressed in *E. coli* (data not shown).

**Figure 5:**
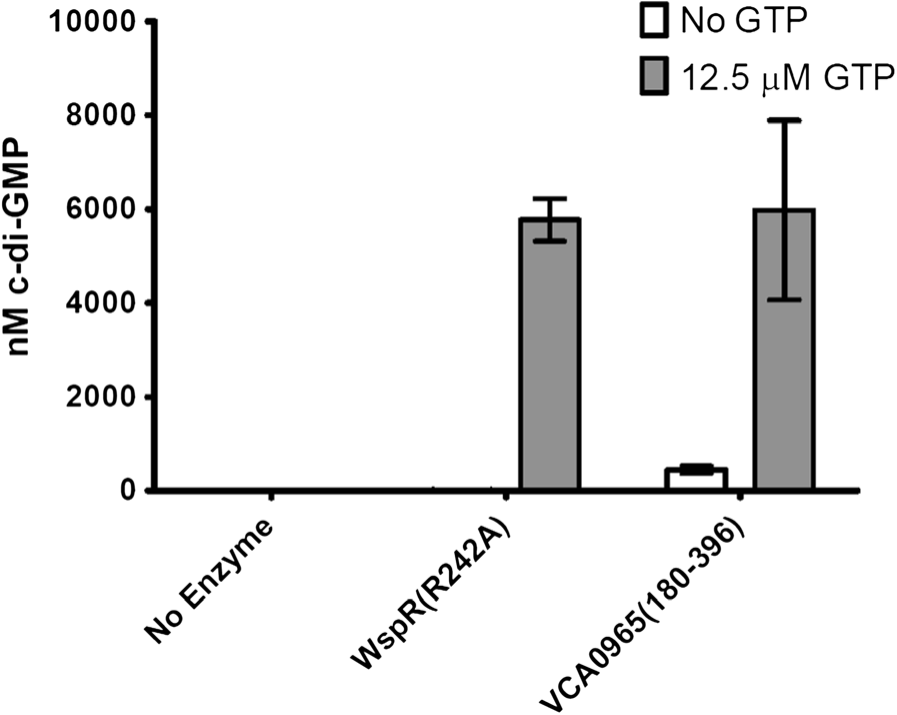
**VCA0965 (180–396) is an active DGC *in vitro*.** The cytoplasmic portion of VCA0965, VCA0965(180–396), was purified. This protein and WspR(R242A) were incubated with and without 12.5 µM GTP overnight. The concentration of synthesized c-di-GMP was determined by LC-MS/MS. The mean value and standard deviation are shown (n = 3).

It remained possible that VCA0965 modulated c-di-GMP levels by interacting with other components in the cell, although these components would have to be conserved between *V. cholerae* and *E. coli*. To further confirm that VCA0965 is an active DGC, we purified VCA0965 and assessed its DGC activity *in vitro*. Due to the fact that VCA0965 is predicted to contain five transmembrane domains that span the inner membrane, it was necessary for purification purposes to construct a truncated version of VCA0965 composed exclusively of the cytoplasmic portion of the protein containing amino acids 180 to 396. VCA0965(180–396) was incubated with 12.5 µΜ GTP and synthesized c-di-GMP was quantified using LC-MS/MS. Both VCA0965(180–396) and the positive control DGC, WspR(R242A) [35], produced c-di-GMP with yields above 90% (Figure 5). LC-MS/MS analysis of VCA0965(180–396) revealed small amounts of c-di-GMP even when GTP was not added. The presence of this c-di-GMP is likely due to co-purification of c-di-GMP bound to the I-site as has been previously observed [36]. The WspR(R242A) positive control is an I-site mutant and thus no co-purification of c-di-GMP was observed. We attempted to define the kinetic parameters of VCA0965 but the purified protein was unstable and frequently precipitated from solution, leading to variable results. This instability is likely because VCA0965 is an inner membrane protein and we were only able to purify its cytoplasmic region. Nevertheless, these results indicate that even though VCA0965 contains a degenerate active site it is capable of c-di-GMP synthesis.

### Determining the plasticity of the first amino acid in the active site of VCA0965

**Figure 6:**
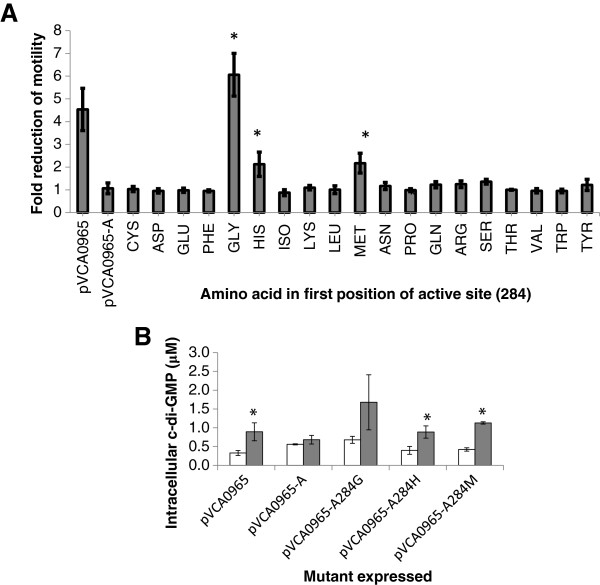
**The active site of VCA0965 tolerates additional amino acid substitutions. (A)** Substitution mutants of every amino acid in the first position of the AGDEF active site of VCA0965 (residue 284) were constructed and examined for the ability to reduce motility of *V. cholerae.***(B)** The intracellular concentration of c-di-GMP from *V. cholerae* expressing WT VCA0965 or the GGDEF, HGDEF, and MGDEF mutants was determined using LC-MS/MS. Open bars are the uninduced conditions while gray bars are induced with 0.1 mM IPTG. Error bars indicate the standard deviation and statistical significance from the corresponding uninduced culture was determined using a one-tailed Student’s t-test (n = 3, *p < 0.05).

As a naturally occurring enzymatically active AGDEF DGC has never been reported, we wondered what amino acid substitutions in the first residue of this active site would maintain DGC activity (amino acid residue 284 in VCA0965). To answer this question, we constructed mutants of VCA0965 containing all 19 amino acids in the first position of the AGDEF active site and determined if expression of these proteins in *V. cholerae* reduced motility (Figure 6A). As expected, mutation of the alanine to glycine generated an active DGC which inhibited motility greater than the WT AGDEF enzyme, although this difference was not statistically significant. Although most VCA0965 mutants lost the ability to inhibit motility, mutation of the alanine to histidine and methionine exhibited a significant inhibition of motility greater than 2-fold.

The activity of the glycine, histidine, and methionine substitutions was further examined by determining if induction of these proteins significantly increased intracellular c-di-GMP in *V. cholerae*. Indeed, all three of these VCA0965 mutants significantly increased c-di-GMP when induced compared to the uninduced controls; the histidine and methionine mutations were statistically significant (p < 0.05 using a one-tailed Student’s t-test) while the glycine substitution bordered on statistical significance with a p-value of 0.067 (Figure 6B). The Ptac promoter used to induce these proteins is leaky accounting for the variation in c-di-GMP levels in the uninduced controls. All four active VCA0965 variants increased c-di-GMP levels by 3-fold. These results indicate that the first position of the VCA0965 active site is somewhat flexible, maintaining activity with four different amino acids.

### Bioinformatic analysis of AGDEF active sites

Our results studying VCA0965 suggest that the AGDEF active site is in fact capable of DGC activity. We determined the prevalence of this active site among GGDEF proteins using the standalone version of NCBI’s PsiBlast to scan the 28,254 sequences of GGDEFs in the Pfam database [37]. We first determined the frequency of the AGDEF active site motif among GGDEF domain containing proteins, and found that approximately 1.6 percent of GGDEFs have an AG(D/E)EF active site motif, approximately half of which also encode a RXXD inhibition site (Table 2). We further found that 23 percent of AG(D/E)EF proteins originate from *Vibrios.* Only about 10 percent of the total GGDEFs derive from *Vibrios* indicating the AG(D/E)EF active site is overrepresented in this genus (Table 2). Finally, we found that the total RXXD motif frequency in GGDEF proteins was 44 percent, lower than the 57 percent frequency reported by Christen *et al.*[19], but this could be due to the large difference in database size (>1000 sequences versus >20,000 sequences).

**Table 2:** Bioinformatic analysis of the frequency of the AG(D/E)EF active site motif

	**Number of proteins**	**Percentage of total GGDEFs**	**Percentage of total AG(D/E)EF proteins**
Total GGDEF domain containing proteins analyzed	28,254	100	n/a
Proteins containing an AG(D/E)EF motif	440	1.56	100
Proteins containing an AG(D/E)EF motif and an RXXD motif	205	0.73	46.59
Proteins containing an AG(D/E)EF motif from *Vibrio* sp.	100	0.35	22.73
Known GGDEF domain containing proteins from *Vibrio* sp.	2946	10.43	n/a
Proteins containing an RXXD and an XG(D/E)EF motif	12410	43.92	n/a

The N-terminal domains of GGDEF proteins typically encode conserved domains that are thought to bind environmental signaling cues to control the associated enzyme domain [38]. HMM-Prot predicts that the N-terminal region of VCA0965 encodes five transmembrane regions with few amino acids present outside of the membrane. To determine the conservation of this domain among GGDEF proteins, we queried the GGDEFs in the Pfam database with the N-terminal region of VCA0965(1–179). Sixty-eight sequences were identified with homology to VCA0965’s N-terminal region, with sixty-two of these proteins containing an AG(D/E)EF active site motif and sixty-three of these proteins originating from *Vibrios* (Table 3). This suggests that this N-terminal domain is found predominantly in related *Vibrios* and is strongly associated with an AG(D/E)EF active site. Moreover, this analysis shows that most of the GGDEFs encoding the AG(D/E)EF active site we identified in Table 2 are not homologs of VCA0965, suggesting other DGCs have evolved to use the AGDEF active site to synthesize c-di-GMP.

**Table 3:** Bioinformatic analysis of the N-terminal region of VCA0965

	**Number of proteins**	**Percentage of total GGDEFs**	**Percentage of total N-terminal analogs**
Proteins containing homology to VCA0965′s N terminus	68	0.24	100
Proteins containing an AG(D/E)EF motif and homology to VCA0965′s N terminus	62	0.21	91.18
Proteins from *Vibrio* sp. containing homology to VCA0965′s N terminus	63	0.22	92.65
Proteins from *Vibrio* sp. containing homology to VCA0965′s N terminus and an AG(D/E)EF motif	61	0.21	89.71

## Discussion

Here we show that VCA0965, which encodes a degenerate AGDEF active site, is an active DGC that synthesizes c-di-GMP. Overexpression of VCA0965 represses motility by increasing c-di-GMP levels in *V. cholerae*, and the DGC domain of VCA0965 is capable of *in vitro* c-di-GMP synthesis. To our knowledge, this is the first description of an active DGC containing a naturally encoded AGDEF active site motif and only the second description of an active non-canonical DGC; the only other example being the SGDEF containing ECA3270 from *Pectobacterium atrosepticum*[26]. ECA3270 regulates Type 1 secretion and was found to retain a reduced level of activity when the active site was mutated to AGDEF. Interestingly, we observed a loss of activity of VCA0965 when the first position was mutation to serine. This suggests that other amino acids in these DGCs have coevolved to tolerate these non-degenerate substitutions in the first position of their active site.

Retention of activity in ECA3270 and VCA0965 is surprising when the steric constraints of the active site are considered. Within the active site, the GGDEF motif is located in a hairpin [39]. C-di-GMP bound at the active site extends over this hairpin, specifically the first G in the GGDEF motif. Although alanine is a conservative change compared to glycine, we were surprised to find that mutation of this alanine to the less conserved histidine and methionine amino acids maintained an active DGC. We would expect that larger amino acids at this position should create steric interactions detrimental to activity. It is not clear why these amino acids are tolerated over less drastic changes, such as valine, but we predict these differences are driven by specific amino acids flanking the active site in proximity to the hairpin. As both ECA3270 and VCA0965 tolerate non-canonical substitutions to the first position of their active sites, but the amino acids that are tolerated appear to be different, the flexibility of each DGC’s active site may follow its own “rules” and caution must be used when determining if noncanonical substitutions in the first residue of a given GGDEF motif render a DGC inactive.

Contrary to what might be expected based on the structure of the DGC domain, degeneracy at the first amino acid is quite common among DGCs. 23.7% of all DGCs are degenerate at one or more amino acids in the GGDEF motif [1]. Of these, almost half vary at only one amino acid and most of these variances occur at the first amino acid [1]. The frequency of variance at the first amino acid of the GGDEF motif despite the apparent steric limitations of the DGC structure suggest that there may be a high level of variance in DGC structure. Our *in silico* bioinformatic analysis of the AGDEF motif found that AG(D/E)EFs account for approximately 1.6 percent of DGCs, making this motif fairly common and suggesting that AGDEFs may have a variant structure that allows them to retain activity. How this variance impacts function remains to be addressed.

Finally, we found that although VCA0965 is able to repress motility and increase intracellular c-di-GMP levels when overexpressed, WT VCA0965 did not impact biofilm formation. However, the lack of biofilm induction is inconsistent as we have found that overexpression of VCA0965 led to an approximately 6-fold increase in biofilm formation in another study [32]. When biofilm formation was measured using flow cytometry, no increase in biofilm formation was observed—a result corroborated by Massie *et. al.*[32]. This inconsistency suggests that at the levels we are inducing VCA0965 it is only able to exert a slight effect on biofilm formation which is highly dependent on experimental conditions. These results are consistent with Massie *et. al.* who observed that some DGCs were able to induce biofilm formation at 12 µΜ c-di-GMP, the levels we determined are produced when VCA0965 is induced (Figure 4), while others did not [32]. This differential induction of phenotypes by distinct DGCs at analogous concentrations of c-di-GMP is attributed to high-specificity signaling pathways. Therefore, it is not surprising for VCA0965 to have an intermediate phenotype. However, removing VCA0965 from feedback inhibition by mutation of the RXXD site increases the activity of VCA0965 allowing it to synthesize higher amounts of c-di-GMP and induce robust biofilm formation when overexpressed.

Our results highlight that the first amino acid of the GG(D/E)EF motif should be considered flexible in regards to DGC activity. In combination with the finding for ECA3270, a more accurate description of a functional active site should be (G/S/A)G(D/E)EF. However, whether enzymes which naturally encode a GG(D/E)EF will retain DGC activity upon substitution of the first glycine to serine or alanine remains to be determined. It is possible that intragenic epistatic interactions are necessary for these alterations of the first amino acid to remain functional. Additional non-canonical active site motifs should be assayed to determine if enzymatic activity of these DGCs is widespread. Furthermore, structural analysis of ECA3270 and VCA0965 can highlight how variance at the first amino acid of the GGDEF motif can accommodate DGC activity.

## Conclusions

The research presented here shows that the AGDEF containing *Vibrio cholerae* DGC, VCA0965, is able to synthesize c-di-GMP to control motility and biofilm formation. Moreover, we found that addition of three other amino acids, glycine, methionine, and histidine, maintained DGC activity. Our results illustrate that the first residue of the GG(D/E)EF active site in DGCs is more flexible to substitutions than previously appreciated. As many DGCs encode non-canonical amino acids at this position, caution must be used when determining the activity of these enzymes. Whether or not substitution of non-canonical amino acids at the first position of the active site has important functional consequences on DGC activity remains to be explored.

## Abbreviations

DGC: Diguanylate cyclase; PDE: Phosphodiesterase; c-di-GMP: Cyclic di-GMP.

## Competing interests

The authors declare no competing interests.

## Authors’ contributions

JLH, GBS and BJK collected and analyzed the data and JLH and CMW wrote the manuscript. All authors read and approved the final manuscript.
